# Genome Editing in *C. elegans* and Other Nematode Species

**DOI:** 10.3390/ijms17030295

**Published:** 2016-02-26

**Authors:** Takuma Sugi

**Affiliations:** 1 PRESTO, Japan Science and Technology Agency, Kawaguchi 332-0012, Japan; 2 Institute for Integrated Cell-Material Sciences (WPI-iCeMS), Kyoto University, Yoshida-Honmachi, Sakyo-ku, Kyoto 606-8501, Japan; tsugi@icems.kyoto-u.ac.jp; Tel.: +81-75-383-2164; Fax: +81-75-383-2541

**Keywords:** nematode, *C. elegans*, transposon, TALEN, CRISPR/Cas9, cell-specific analysis

## Abstract

*Caenorhabditis elegans*, a 1 mm long free-living nematode, is a popular model animal that has been widely utilized for genetic investigations of various biological processes. Characteristic features that make *C. elegans* a powerful model of choice for eukaryotic genetic studies include its rapid life cycle (development from egg to adult in 3.5 days at 20 °C), well-annotated genome, simple morphology (comprising only 959 somatic cells in the hermaphrodite), and transparency (which facilitates non-invasive fluorescence observations). However, early approaches to introducing mutations in the *C. elegans* genome, such as chemical mutagenesis and imprecise excision of transposons, have required large-scale mutagenesis screens. To avoid this laborious and time-consuming procedure, genome editing technologies have been increasingly used in nematodes including *C. briggsae* and *Pristionchus pacificus*, thereby facilitating their genetic analyses. Here, I review the recent progress in genome editing technologies using zinc-finger nucleases (ZFNs), transcriptional activator-like nucleases (TALENs), and clustered regularly interspaced short palindromic repeats (CRISPR)/Cas9 in nematodes and offer perspectives on their use in the future.

## 1. Introduction

The nematode *C. elegans* became a powerful model organism just over 50 years ago [1]. This tiny animal has greatly contributed to investigations of the functions of genes in development and cellular biology. One advantage of using *C. elegans* as a model animal is its simple morphology; the adult hermaphrodite is composed of only 959 somatic cells, including 302 neurons, and cell number does not vary among individuals. Researchers have tracked the fate of every cell, from fertilization to adulthood, in live animals and have generated a complete cell lineage [2]. This insight into cell lineage has contributed enormously to research in the field of developmental biology. The wiring and connectivity of neurons in *C. elegans* have been completely reconstructed from electron micrographs [3]. Therefore, this well-defined nervous system simplifies investigations of the neural circuitry underlying behavioral plasticity. Features that make the nematode highly amenable to laboratory research also include its small size, short life cycle and ability to survive long-term freezing for storage. These advantageous features have allowed *C. elegans* researchers to uncover novel biological mechanisms, such as apoptosis, and develop novel techniques, such as green fluorescent protein (GFP) tagging, in the life sciences research field.

*C. elegans* is a genetically tractable model system, for which many genetic resources and tools have been developed. There are approximately 20,359 protein-coding genes in *C. elegans* (WormBase referential freeze WS250, November 2015). In OrthoList, a compendium of *C. elegans* genes with human orthologs, 7663 protein-coding genes are predicted to have human orthologs, corresponding to approximately 38% of the *C. elegans* genome [4]. Furthermore, 60%–80% of human genes are represented by an ortholog in the *C. elegans* genome [5]. More importantly, 40% of genes known to be associated with human diseases are represented by orthologs in the *C. elegans* genome [6]. 

To clarify functional roles of *C. elegans* genes, various genetic methods have been developed and adapted before emergence of recent genome engineering techniques. In forward genetic screening, worms are treated with mutagens such as ethyl methane sulfonate (EMS) to induce DNA lesions and mutants with a target phenotype. Subsequent gene mapping and detailed analyses of the mutant phenotype allow elucidation of the gene’s function. As a complement to forward genetics, reverse genetics offers opportunities to examine the function of a gene by analyzing the phenotypic effects of targeted gene sequence alteration. RNA interference (RNAi) has served as a powerful reverse genetic tool for large-scale genetic screening and to study gene function by examining the consequences of targeted gene knock-down. However, RNAi fails to completely suppress gene expression and provides only temporally inhibition of gene function only temporally. Screening of worms with deletions of specific target DNA by the *C. elegans* Gene Knockout Consortium and the Japan National Bioresource Project has offered opportunities to examine the phenotypic effects of a targeted gene knock-out. However, these resources do not completely include mutants with deletions in a desired genomic region. Therefore, it has been needed to establish a strategy for inducing targeted gene knock-out at will. 

In compatible with forward and reverse genetic screening, microinjection of a gene of interest has been an indispensable genetic technique to study gene functions in *C. elegans*. The microinjection of genes including fluorescent protein-coding genes into the gonad of the adult hermaphrodite results in the formation of semi-stable extrachromosomal arrays that are comprised of many copies of the injected DNA [7]. The microinjection under the control of a cell-specific promoter has been used for an extrachromosomal rescue experiment that enables identification of a cell-specific role of the transgene. The transgenes, which are typically overexpressed in somatic tissues and silenced in the germline and early embryo, can be integrated into the genome by UV (ultraviolet) irradiation or microparticle bombardment [8,9]. These integration methods have allowed the generation of low-copy transgenes, which are expressed at closer to endogenous levels [10]. However, this experiment is less efficient, time-consuming, and not easily able to control genomic location for gene insertion. Thus, a strategy for inducing a stable gene knock-in at a desired genomic location has been required in the nematode research field.

Due to these requirements for targeted genome editing, *C. elegans* geneticists have developed methods based on the *Mos1* transposon system [11,12]. Then, zinc-finger nucleases (ZFNs) [13], transcription activator-like effector (TALE) nucleases (TALENs) [14,15], and RNA-guided clustered regulatory interspaced short palindromic repeats (CRISPR)/Cas9 endonuclease systems have emerged as the revolutionized techniques adapted for creating locus-specific mutations in nematodes [15,16,17,18,19,20,21,22]. In this review, I provide a historical overview of this research field and discuss recent implementations of genome editing techniques in nematodes. 

## 2. Development of Gene Editing Protocols for Nematodes

### 2.1. Site-Restricted Editing by Mos1

Before the revolution of the current genome editing technologies, methods based on the *Mos1* transposon system allowed for targeted genome editing in *C. elegans* [11,12]. Robert and Bessereau reported that the *Drosophila* element *Mos1* can also be used to produce double strand breaks (DSBs) and initiate gene editing [11]. In the experiments described in that paper, a repair template was constructed by preparing a transgene containing sequences homologous to the target genome region. The repair template was co-injected into the germline of a *Mos1*-insertion mutant with a vector enabling the expression of the *Mos* transposase using the heat-shock promoter *hsp-16.48*. Heat-shock treatment triggered DSBs at the target site, which were repaired by transgene-mediated gene conversion. This process allowed for mutations in the transgene to be copied at a specific locus at frequencies ranging from 10^−5^ to 10^−4^ events per F1 progeny. The mutations were successfully inherited by offspring. Frøkjær-Jensen *et al.* developed methods for *Mos1*-mediated single-copy insertion (MosSCI) that enables the insertion of single copies of transgenes into well-defined genomic loci [12] and optimized the efficiency of insertion [23]. The prerequisite for these techniques was the insertion of the *Mos1* element into the target genomic region. Although efforts to generate a comprehensive *Mos1*-inserted mutant library are in progress, the ability to modify the genome with this technique is limited to genetic loci near transposons.

### 2.2. Universal Editing with ZFNs, TALENs and CRISPRs

Early methods for artificially generating DSBs at targeted genomic regions were based on ZFNs. ZFNs are chimeric proteins composed of DNA-binding Cys_2_His_2_ zinc finger motifs and a nonspecific FokI nuclease domain. Each finger is about 30 amino acids and interacts with a separate DNA triplet. Both natural and artificial zinc fingers have been characterized and known to bind to all 5’-GNN-3’, many ANN and CNN, and some TNN triplets. In addition, the modular nature of the zinc fingers enables them to be assembled in arbitrary combinations. In general, three zinc fingers are combined to recognize a specific 9 bp DNA sequence with nanomolar affinity, and additional fingers can be used to increase specificity. DSBs require the dimerization of two FokI nuclease domains, and ZFNs are used in pairs with specificity to opposing DNA strands, which enables assembly on both sides of the targeted site. This property enhances the specificity of ZFN targeting. Because zinc fingers exist for many of the DNA triplets, varying the number and types of fingers in each chimera would theoretically allow for targeting nucleases to the desired genomic loci. Carroll and colleagues initially designed a synthetic target DNA sequence for a previously characterized ZFN [13]. The synthetic target DNA along with the plasmid expressing the nuclease with a heat-shock promoter were co-injected into germline cells to generate an extrachromosomal array. Expression of the nuclease by heat-shock treatment induced mutations in more than 20% of the target sites. In subsequent experiments, an endogenous genomic sequence was targeted using a pair of designed ZFNs. This experiment achieved mutations in approximately 20% of the target sites by heat-shock induction. This study was the first to demonstrate that custom-engineered ZFNs could effectively generate targeted DSBs in nematode DNA. Although the repair of these breaks by the end-joining mechanism often generated mutations at the targeted locus, the procedure had little practical value as a genetic tool because the mutations were generated in somatic cells and hence were not inherited by offspring. Thus, these studies did not achieve the goal of heritable genome editing using ZFNs. The authors proposed that direct injection of mRNA into the germline might be effective for producing inherited mutations.

In a landmark paper on genome editing released in 2011, Wood *et al.* demonstrated heritable genome editing using ZFNs in both *C. elegans* and *C. briggsae* [14]. In the experiments described in this paper, the endogenous gene *ben-1* was initially targeted because the phenotype of *ben-1* heterozygous mutants can be scored easily by analyzing mobility on the paralysis-inducing drug benomyl. To attain heritable mutations in *C. elegans*, the investigators set the precedent of using mRNA, rather than DNA, delivery for getting germline production. As a proof of principle, the authors injected gonads with ZFN-encoding mRNAs carrying 5’ and 3’ untranslated regions amenable to germline translation and obtained *ben-1/+* mutants at a frequency of approximately 3% in F1 progeny. To establish a strategy for identifying mutants by molecular genotyping rather than relying on a visible phenotype, they targeted the REX-1 locus; REX-1 recruits the dosage compensation complex (DCC), thereby reducing gene expression. Using the CEL-1 assay, the authors isolated 18 independent mutant lines from 338 CEL-1 reactions, indicating that molecular genotyping was an effective method for phenotypic selection.

In TALENs, a nonspecific FokI nuclease domain is fused to a customizable TALE domain which contains tandem repeats of 33 or 34 amino acid segments and recognizes a predictable DNA sequence [24]. Residues 12 and 13 of the segment, referred to as repeat variable diresidues (RVDs), determine the base-specific binding (HD to C, NI to A, NG to T, NN to G) [25,26]. Sequence-specific DNA recognition by large TALE domains directs the nuclease, and the nuclease introduces DSBs at a prospective target site. Erroneous repair by end-joining often yields a mutagenic deletion or insertion at this breakpoint [27]. Compared with that of ZFNs, the recognition sequence of TALEs is relatively simple, and TALEs can be designed to bind to almost any sequence. Moreover, TALENs also bind as pairs, which also enhances specificity like ZFNs do. These features of TALEs are advantageous for many researchers who use genome editing techniques.

Woods *et al.* first utilized TALENs in nematodes as reported in their paper describing ZFNs [14]. TALEN-encoding mRNAs carrying 5’ and 3’ untranslated regions designed to target the *ben-1* gene were injected into the *C. elegans* germline [14]. This method resulted in many TALEN-induced *ben-1* mutations at a frequency of 1.1% in F1 progeny, and the same approach has been applied to *C. species 9* (now called *C. nigoni*; 2.4% frequency) and *P. pacificus* (6.8% frequency) as described in a subsequent paper [15]. Wei *et al.* have also used TALENs in *C. briggsae* and *C. tropicalis* [28,29]. The isolation of targeted mutants in *C. elegans* has typically relied on visible phenotypes or fluorescent markers such as GFP. In addition, molecular genotyping using CEL-1 assays [14], polyacrylamide electrophoresis [28], and heteroduplex mobility assays [30] has also been used to identify mutations that are not associated with a visible phenotype.

Although genome editing using TALENs has almost completely overcome the limitations associated with some target sequences, the technique requires the laborious custom design of TALE repeat segments. The CRISPR/Cas9 system emerged as a conceptually simple method that can be used as an alternative to TALENs [31,32,33]. In bacteria and archaea, the CRISPR locus functions with Cas proteins to facilitate an adaptive immune system response against invading foreign DNA, such as viral DNA or plasmids. The foreign nucleotides are incorporated into the CRISPR locus in the host genome, which results in short CRISPR RNAs (crRNAs) that direct the sequence-specific cleavage of homologous target dsDNA by Cas endonucleases. Previous studies with the type II CRISPR/Cas system, which requires the nuclease Cas9, a targeting crRNA and an additional trans-activating crRNA, have revealed that fusing the targeting and trans-activating RNAs to form a single guide RNA (sgRNA) is sufficient to direct Cas9-mediated target cleavage [31]. This system has been used for genome editing in other model organisms because of the simplicity in designing a sgRNA. 

Friedland *et al.* initially constructed a plasmid that drives the expression of the gene encoding Cas9 using the germline-specific *eft-3* promoter [16]. In that paper, *klp-12* and *Y61A9LA.1*, genes with an unknown loss-of-function phenotype, were targeted. The researchers isolated *klp-12* and *Y61A9LA.1* mutations in 80.3% and 18.1% of the F1 worms screened, respectively. With respect to the *klp-12* locus, 27 of 80 F1 worms carried a homozygous mutation. This mutation frequency is high enough to preclude the requirement of an obvious phenotype or visible selection marker. Like the TALEN system, the CRISPR system has been also proven to be useful for researchers working with non-*Caenorhabditis* nematodes. Witte *et al.* have successfully used CRISPR system-mediated gene inactivation in *P. pacificus* [34]. Thus, the CRISPR system is undoubtedly a versatile method to facilitate genome editing in the nematode.

Other research groups have also developed useful protocols and have demonstrated that heritable changes in nematode genes can be induced using the CRISPR/Cas9 system [15,17,18,19,20,21,22,35]. The protocols used in these experiments primarily differ with respect to delivery of the CRISPR/Cas9 effector complex (DNA, RNA, or protein) and the methods used to identify mutations (PCR screen, visible markers, or obvious phenotypes). For these experiments, most of the research groups expressed Cas9 under the control of the *eft-3* promoter. On the other hand, Boxem’s group found the possibility that expression of Cas9 from the *eft-3* promoter causes embryonic lethality. The authors expressed Cas9 using a heat-shock promoter within a limited time frame to prevent the lethality effects observed with the use of the *eft-3* promoter and indicated that the use of the heat-shock promoter for Cas9 expression might be better than that of the *eft-3* promoter [21]. Other laboratories have used Cas9 and sgRNAs transcribed *in vitro* [17,19] or crRNAs and tracrRNAs transcribed separately *in vitro* [15]. In one unique protocol, similarly to the RNAi feeding strategy, sgRNA was delivered to worms via bacterial feeding to achieve gene disruptions in a time- and labor-saving manner [36]. In this protocol, worms carrying *pie-1p::Cas9* DNA were fed HT115 bacteria transformed with sgRNA. The mutation rates observed for the *bli* and *dpy* loci in this feeding protocol were low compared with the injection method, at 1.40% and 0.92%, respectively. However, the authors have proposed that the protocols could potentially be redesigned to enable large-scale studies using a worm sgRNA library.

In brief, current ZFN, TALEN and CRIPSR systems have made possible important advances, but each has advantages and disadvantages in genome editing. In Zinc fingers, the context-dependency of the binding preference is that Zinc fingers exhibit context-dependent binding effects because of interactions between adjacent modules assembled into a larger array. In addition, assembly of functional ZFNs with the desired DNA-binding specificity remains a laborious process that requires extensive cell-based screening. In contrast, because TALE repeat domains exhibits fewer context-dependent preferences, researchers can simply assemble them in a modular fashion using a simple one-to-one code between individual repeats and the four possible DNA nucleotides. Frequency of off-target effects of TALENs is relatively low because of the length of the target sequence (15–19 bp), the higher specificity of the TALE DNA-binding domain, and the need of FokI dimerization to reconstitute endonuclease activity. On the other hand, despite these advantages over ZFNs, construction of novel TALE arrays still remains labor-intensive partly due to their repetitive sequences. In terms of target design simplicity, sgRNAs can be designed readily and cheaply to target nearly any genomic sequence specifically. Furthermore, the efficiency of the CRISPR system is high compared with the TALEN system. However, multiple mismatches between the guide RNA and its complementary target DNA sequence can be tolerated, thereby causing undesired off-target mutagenesis. Recent studies have demonstrated in other organisms that the use of dual nickases for Cas9 could reduce the potential off-target mutagenesis. Therefore, application of this strategy further revolutionizes the genome editing technology in the nematodes.

### 2.3. Imprecise and Precise Repair Using ssOligo and dsDNA

Genome editing techniques are a promising method for introducing DNA sequences encoding GFP or other tags and large-scale deletions at DSB sites. The methods for knock-in largely rely on the precise homology-dependent repair (HDR) of DSBs, in which a donor template that carries homology arms can be integrated during the repair process. Single-stranded oligonucleotides (ssOligos) and double-stranded DNA (dsDNA) including PCR fragments and plasmid DNAs, all of which contain homology arms, have been used as HDR donor templates [37]. ssOligos and PCR fragments can be easily designed and synthesized within a few days, and their use facilitated HDR-mediated knock-in. On the other hand, plasmid DNAs require cloning but can accommodate gene-sized edits and longer homology arms during repair processes. A previous evaluation of donor templates indicated that ssOligos are the preferred template in *C. elegans*, owing to their efficiency as well as ease of use (average frequencies for ssOligos and dsDNAs are 52% and 20%, respectively) [38]. In addition to HDR, other repair mechanisms also serve as inducing knock-ins and deletions at the targeted genomic loci. Van Schendel *et al*. have described an unexpected finding that while somatic cells use non-homologous end-joining (NHEJ), germ cells use exclusively polymerase theta-mediated end-joining (TMEJ), a simple repair mechanism requiring only one nucleotide as a repair template [39]. The researchers have demonstrated that polymerase theta is solely responsible for the vast majority of insertions and deletions that occur during the natural evolution of *C. elegans*. In addition, the imprecise end-joining repairs as well as template-mediated imprecise and precise HDRs have induced deletion mutations of variable length at the site of the DSB. 

Lo *et al.* have demonstrated for the first time that TALENs can be used to introduce precise changes at endogenous sites using HDR templates [15]. This study was the first evidence that engineered nucleases and exogenous ssOligos as repair templates could be used to achieve heritable changes consisting of single nucleotide changes or insertions (e.g., HA tag) through precise HDR in nematodes at frequencies ranging from 0.2% to 4.0%. Additionally, FLP recombinase was introduced into the strains possessing FRT sites flanking a *cis*-acting regulatory motif to knock out ~1 kb of DNA, resulting in the removal of all the *cis*-regulatory information. By using an ssOligo, Lo *et al.* also demonstrated precise insertion of the HA tag gene in *P. pacificus* (1.0% frequency). These results were the first to demonstrate tagging of an endogenous gene in a manner leaving no detectable marks other than the tag.

**Figure 1:**
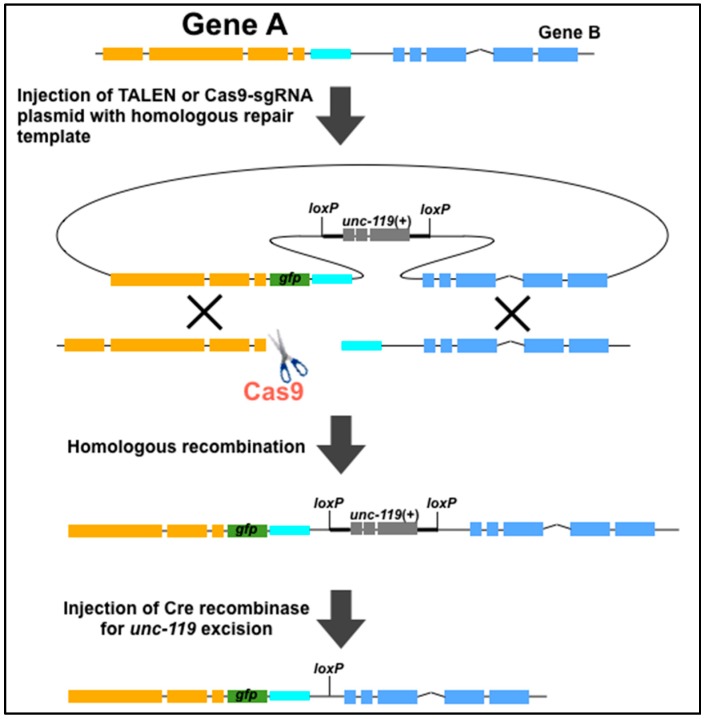
Schematic of homologous recombination-mediated *gfp* insertion using TALENs or Cas9. The *unc-119(+)* selection marker in the resulting *gfp*-inserted worms can be excised by injecting Cre recombinase. The orange, blue, and cyan genomic regions are gene A, the 3’ UTR of gene A, and gene B, respectively. The figure was adapted from Dickinson *et al.* [40].

The CRISPR/Cas9 system has been also used with HDR templates. Dickinson *et al.* has generated HDR-mediated in-frame *gfp* insertions and targeted mutations with an average frequency of 22% [40]. The authors constructed an HDR template comprising the C-terminal 1.5 kb sequence of the *nmy-2* gene (illustrated as Gene A in Figure 1) fused in-frame to *gfp*, the *nmy-2* 3’ UTR, and the *unc-119*(+) selection marker along with 1.5 kb of downstream genomic sequence (Figure 1) [40]. The *unc-119*(+) gene was flanked by loxP sites, which allowed for the excision of this gene by expression of Cre recombinase. At nearly the same time, Katic *et al.* also demonstrated transgene-induced gene conversion of the *daf-2(m579)* allele through Cas9-induced DSB repair by a wild-type sequence [19]. The investigators constructed a sgRNA targeting a 20 bp sequence in *daf-2* that overlapped with the *m579* mutation. The sgRNA was co-injected with a plasmid bearing the 715 bp wild-type *daf-2* sequence, which causes a missense change from *m579* into the wild-type sequence [19]. Instead of the plasmid, ssOligos and PCR fragments with short (~30–60 bases) homology arms was previously examined as a donor template [41]. This micro-homology arm also enabled gene conversion and generated precise nucleotide changes via the CRISPR/Cas9 system. Furthermore, a variety of sequences encoding protein tags, such as V5, 3xFlag, Myc, and OLLAS, with short homology arms can be inserted into the desired target site through precise HDRs [42].

In terms of deletion experiments, Lo *et al.* showed that deletions can be induced either immediately adjacent to the DSB by the end-joining mechanism (2.5% frequency) or distant from the DSB by HDR (13.1% frequency) using TALEN [15]. In another report, co-injection of worms with dual sgRNAs and Cas9 DNA has successfully induced the deletion of a chromosomal segment at least 24 kb in length [43]. Paix *et al.* have also reported that large deletions can be created directly by end-joining mechanism using Cas9 and sgRNA pairs targeting the 5’ and 3’ ends of *mbk-2* and the *swan-1/swan-2* operon at 2.8% and 1.6% efficiencies, respectively [42]. Authors then examined whether ssOligos could be used to precisely create gene-length deletions by HDR and attempted to delete an entire ORF using an ssOligo targeting the K08F4.2 gene containing 51–80 bp micro-homology arms that flanked the sgRNA excision sites. Using this technique, they succeeded in generating large deletions with precise breakpoints in the K08F4.2 gene at a frequency of 3.3%.

## 3. Optimization of the CRISPR System

Along with developments using the CRISPR system, several strategies for efficient identification of genome-edited worms have also been established in *C. elegans*. One such strategy is antibiotic resistance selection. Resistance to hygromycin B conferred by the HygR gene provides an efficient selection method in *C. elegans*. Thus, Chen *et al.* have combined this selection method with the CRISPR system and have created a HygR cassette flanked by ~2 kb of DNA sequence homologous to either side of *ben-1a* sites [35]. CRISPR-based site-directed insertion of the HygR gene enabled hygromycin B-resistant selection of the desired *ben-1* mutants. A toolkit composed of template-mediated repair cassettes that contain both an antibiotic resistance gene and a fluorescent visual maker, which facilitates identification of worms carrying the repair template, has also been developed [44]. 

Although these methods have facilitated the identification of specific mutants, a strategy that did not involve introducing an exogenous sequence at the targeted locus or marker mutations was still desired. Therefore, Dickinson *et al.* have designed a plasmid that includes both a fluorescent tag gene and the self-excising cassette (SEC) carrying a drug resistance gene, a visible phenotypic marker, and a heat-shock-inducible Cre recombinase gene [45]. Incorporation of the SEC from the plasmid into genomic loci permits easy excision of unwanted sequences upon heat-shock treatment after identification of the desired mutant using drug selection and screening for a visible phenotype.

**Figure 2:**
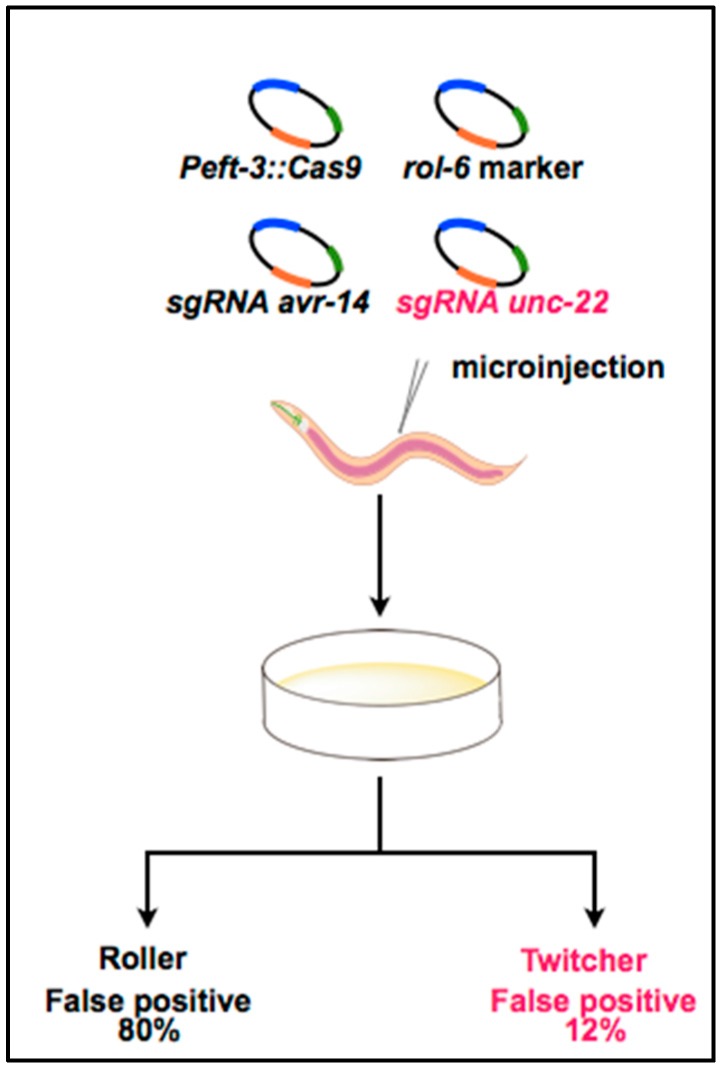
Overview of the co-CRISPR strategy for efficiently identifying functional sgRNAs targeting *avr* genes. sgRNA targeting *unc-22*, a gene associated with the twitcher phenotype, was co-injected with sgRNAs targeting *avr-14* and *avr-15*, the Cas9 expression vector, and the *rol-6* transformation marker. The co-injection of a functional *unc-22* sgRNA reduced the false-positive rate in the identification of mutants. Figure 2 was adapted from Kim *et al.* [46].

Mello and colleagues have reported a “co-CRISPR” strategy that facilitates the identification of functional sgRNAs and increases the yield of transgenic worms carrying an end-joining or homologous recombination event (illustrated in Figure 2) [46]. The authors have found that approximately half of the sgRNAs tested are not effective in practice. This result is partly because conventional markers such as *rol-6* result in false-positive mutant identification, and many injections fail to produce mutant progeny, even if high-quality reagents are used. Therefore, as an efficient marker, Mello *et al.* used an sgRNA targeting the muscle structural gene *unc-22*, which has been previously proven to be active in generating CRISPR/Cas9-induced end-joining mutations, leading to an easily observable paralyzed twitching phenotype [47]. The selection based on the proven end-joining-induced phenotype would facilitate efficient identification of animals in which the CRISPR system was active. To examine this co-CRISPR strategy, the researchers co-injected the *unc-22* sgRNA with targeted sgRNAs for *avr-14* and *avr-15* or for *pie-1b* and *pie-1c*. Interestingly, selection based on the twitching phenotype dramatically increased the yield of animals in which a targeted sgRNA was active.

Arribere *et al.* have established a dominant phenotypic oligonucleotide template conversion strategy [48]. In this strategy (referred to as co-conversion strategy), instead of the sgRNA that induces end-joining-mediated mutations, an sgRNA and donor oligonucleotide to create the dominant phenotypic mutation such as *rol-6*(*su1006*) are co-injected with an sgRNA and donor oligonucleotide to create a mutation in the desired gene. By identifying the desired mutation among F1 progeny heterozygous for the dominant marker mutation, F2 worms that have lost the marker mutation can be isolated with the desired mutation in an unmarked genetic background. Arribere *et al.* have also identified one mutation in *dpy-10*(*cn64*) and one mutation in *sqt-1*(*e1350*) as effective dominant markers. Recently, the repair of a temperature-sensitive lethal point mutation has been used as an alternate co-conversion marker [49]. This strategy promises robust selection, minimal screening, and has no requirement for out-crossing marker mutations. 

In addition to these streamlined screening methods, another group has described the optimization of CRISPR target choices. A new sgRNA guide design strategy that vastly improved the efficiency of editing with Cas9 has been established [50]. The strategy uses sgRNA with a 3’ GG addition to the target-specific sequence, which is key for improving the frequency of mutagenesis. When paired with the co-CRISPR approach, this technique offers the most reliable method of achieving precise genome editing in nematodes.

The CRISPR-mediated HDR protocols have also been improved in another manner. As an alternative to conventional protocols, the direct injection of recombinant Cas9 protein complexed with guide RNA into *C. elegans* induces targeted gene disruption and yields HDR-mediated edits with frequencies ranging from 3.3% to 9.4% [18,51]. This strategy obviates the need to optimize DNA sequences for efficient expression of Cas9 and guide RNAs. Combined with the co-CRISPR strategy, Cas9/sgRNA ribonucleoprotein complexes have been proven more efficient and robust for use with low-efficiency guide RNAs and generating complex edits [51]. The author has established a direct-delivery protocol that increases the number of injected animals that produce mutant progeny (identified at frequencies ranging from 2% to 70% of F1 progeny), representing a 10-fold improvement over conventional plasmid-based protocols.

## 4. Use of Genome Editing Tools to Create New Methods for *C. elegans*

In *C. elegans*, genetic studies can be conducted in a cell-specific manner. This advantage enables the conditional editing of a targeted sequence in the *C. elegans* genome. Conditional editing allows for the examination of genes associated with an embryonic lethal phenotype. Recently, Cheng *et al.* have developed a novel method to generate conditional knock-outs by expressing TALENs in somatic cells (referred to as somatic TALENs) [52]. The authors targeted the *cor-1* gene, a worm ortholog of mouse actin-binding protein coronin. A *cor-1* null mutation causes embryonic lethality, and its function was not known. The authors revealed that expression of a somatic TALEN targeting the *cor-1* under the Q-cell-specific promoter causes the migration defects of the AQR and PQR neurons that descended from the Q-cell in *C. elegans* larva, indicating the role of the *cor-1* gene in Q-cell development. Thus, conditional knock-out was established by germline transformation with plasmids encoding TALENs using a cell-specific or inducible promoter. A similar strategy has also been established with the CRISPR system under the control of other cell-specific promoters. This strategy has helped to gain a further mechanistic insight into *cor-1* [53] and to uncover roles of several other embryonic essential genes, including the dynein intermediate chain and a cytokinetic scaffold protein Anillin [36,54,55]. 

In terms of conditional protein depletion, more recently, the auxin-inducible degradation (AID) system discovered in plants was applied to *C. elegans* [56]. It has been known through other model organisms that expression of an *Arabidopsis* TIR1 F-box protein mediates robust auxin-dependent degradation of degron-tagged targets by proteasomes. As proof of principle, the expression of TIR enabled rapid, reversible, auxin-dependent depletion of nuclear and cytoplasmic targets in diverse somatic and germline tissues throughout development in *C. elegans*.

Recently, Yumerefendi *et al.* have used Cas9-mediated homologous recombination with an interesting twist: a gene encoding light-activated protein was used as a repair template in place of tagged proteins [57]. The authors inserted a gene for light-activated nuclear shuttle proteins (referred to as LANS), which can change their nuclear localization upon light stimulation, into the *C. elegans* *lin-1* locus via Cas9-mediated homologous recombination. A light-dependent functional switch of endogenous cellular transcription factor enabled optogenetic control of vulval cell fate specification in *C. elegans*.

CRISPR interference (CRISPRi), which has been developed to study sgRNA-mediated sequence-specific repression of transcription in prokaryotic and eukaryotic cells, was applied to *C. elegans* and *D. rerio* [58]. Long *et al.* revealed that transcription initiation and elongation of a gene can be interfered by the presence of the gRNA:DNA heteroduplex/dCas9 (a catalytically inactive form of Cas9) complex in its promoter and exons [58].

## 5. Important Results Achieved Using Genome Editing Techniques

Genome editing techniques have helped to identify biological functions of genes in *C. elegans* [56,59,60,61]. TALEN-mediated genome editing has been used for the targeted inactivation of the *paxt-1* gene, which encodes a subunit of the XRN2 complex which is a eukaryotic exoribonuclease for processing and degrading various substrates [61]. This inactivation reduced XRN2 protein levels, decreased miRNA turnover, and caused lethality, suggesting that stabilization of XRN2 is a major function of PAXT-1. 

Using gene editing, Ellis’s group has addressed the molecular mechanisms underlying the evolution of self-fertile nematodes [29,62]. The investigators generated knock-out mutants of key genes involved in sperm activation in *C. briggsae* or *C. tropicalis* and found that *C. briggsae*, *C. elegans*, and *C. tropicalis* become self-fertile by co-opting one of the two redundant male programming pathways: the SPE-8 tyrosine kinase pathway or the TRY-5 serine protease pathway. These pathways functioned redundantly in ancestral males [29]. Characterizations of an identified *spe-47*(*hc198*) allele using the CRISPR system showed that the *hc198* mutation is able to bypass the SPE-8 signaling pathway to cause premature activation of a small fraction of sperm [63]. In addition, the authors have found that the nucleosome remodeling factor (NURF) complex has acquired a unique role in the sperm/oocyte decision of *C. briggsae* [62]. This role was acquired during recent *C. briggsae* evolution. 

Meyer and colleagues have used the CRISPR system to investigate X chromosome dosage compensation, which balances gene expression between XX hermaphrodites and XO males [59]. Crane *et al.* used genome-wide chromosome conformation capture techniques to obtain three-dimensional (3D) maps of the *C. elegans* genome [59]. They initially revealed that the DCC remodels X chromosomes into a sex-specific spatial conformation distinct from autosomes. The dosage-compensated X chromosomes had been known to consist of self-interacting domains similar to topologically associated domains (TADs) in mammals. To address whether DCC-dependent interactions between sequence-specific recruitment elements on X (*rex* sites) create TAD boundaries, they deleted the endogenous *rex-47* site from a DCC-dependent TAD boundary using the CRISPR system. This experiment shows that the DCC contributes to inducing and reinforcing TAD boundaries on the X chromosome by mediating long-range interactions between its highest affinity *rex* sites. This critical finding led the authors to conclude that the DCC dynamically remodels X chromosomes into a spatial conformation of TADs.

In addition to these achievements, genome editing has been a practical approach for investigating the role of microRNAs in vulval development [60]. Ecsedi *et al.* have found through editing the endogenous *let-7* target site that, although *let-7* has numerous targets including LET-60/RAS, it does not function to reduce gene expression noise broadly but instead functions to direct vulval development by regulating a single primary target, LIN-41/TRIM71 [60]. In terms of behavioral mechanism, Uozumi *et al.* found through proteomic screening that the mitochondrial voltage-dependent anion channel VDAC-1 functions as a candidate target of MAPK, and uncovered its role in olfactory behavior using the CRISPR system [64]. Thus, the practical application of genome editing techniques has facilitated the identification of novel roles for genes that had not been previously investigated by genetic experiments.

## 6. Conclusions

In nematode species, as with other model organisms, genome editing technologies have been used as indispensable tools for investigating biological systems. The previously available methods to generate mutations, such as chemical mutagenesis, use laborious procedures that require at least one month for large-scale mutagenesis screening, with unpredictable results. In contrast, recently developed genome editing techniques allow researchers to produce specific knock-out mutants or missense mutants in two to three weeks. Furthermore, they can conditionally create the targeted mutations with a cell-specific promoter. This technique provides an alternative avenue to investigate the role of a gene of interest in a cell-specific manner, avoiding the embryonic lethality caused by some mutations. 

In other model organisms, TALE and Cas9 can be altered into a transcriptional regulator when they are fused to the transcriptional activator VP16/VP64 [65,66] or to an epigenetic factor such as LSD1 histone demethylase [67]. Moreover, as an alternative to fluorescence *in situ* hybridization (FISH) methods, fluorescently tagged Cas9 labeling of specific DNA loci has been developed as a powerful live-cell imaging strategy [68]. These techniques should be highly compatible with *C. elegans* genetic methods, in which genes are expressed in only a target cell with a cell-specific promoter. Considering this remarkable feature, the causal roles of a gene expression change and its associated chromatin state at the single cell level can be studied in diverse biological processes including development, learning and memory, and disease. In summary, further promising techniques and novel biological concepts should emerge from the combination of genome editing technology and nematode cell-specific genetics.
